# Behavioral and Neuronal Effects of Inhaled Bromine Gas: Oxidative Brain Stem Damage

**DOI:** 10.3390/ijms22126316

**Published:** 2021-06-12

**Authors:** Shazia Shakil, Juan Xavier Masjoan Juncos, Nithya Mariappan, Iram Zafar, Apoorva Amudhan, Archita Amudhan, Duha Aishah, Simmone Siddiqui, Shajer Manzoor, Cristina M. Santana, Wilson K. Rumbeiha, Samina Salim, Aftab Ahmad, Shama Ahmad

**Affiliations:** 1 Department of Anesthesiology and Perioperative Medicine, University of Alabama at Birmingham, Birmingham, AL 35294, USA; shaziaahd8@gmail.com (S.S.); jxjuncos@uabmc.edu (J.X.M.J.); nmariappan@uabmc.edu (N.M.); iramzafar@uabmc.edu (I.Z.); apoorva7215@gmail.com (A.A.); archita0315@gmail.com (A.A.); duha.aishah@gmail.com (D.A.); ssiddiqui@uabmc.edu (S.S.); smanzoor@uabmc.edu (S.M.); aftabahmad@uabmc.edu (A.A.); 2 Veterinary Diagnostic and Production Animal Medicine, Iowa State University, Ames, IA 50011, USA; csantana@iastate.edu; 3 Department of Molecular Biosciences, School of Veterinary Medicine, University of California, Davis, CA 95616, USA; wkrumbeiha@ucdavis.edu; 4 Department of Pharmacological & Pharmaceutical Sciences, University of Houston, Houston, TX 77004, USA; ssalim2@central.UH.edu

**Keywords:** bromine, halogens, injury, brain, behavior, neuronal

## Abstract

The risk of accidental bromine (Br_2_) exposure to the public has increased due to its enhanced industrial use. Inhaled Br_2_ damages the lungs and the heart; however, adverse effects on the brain are unknown. In this study, we examined the neurological effects of inhaled Br_2_ in Sprague Dawley rats. Rats were exposed to Br_2_ (600 ppm for 45 min) and transferred to room air and cage behavior, and levels of glial fibrillary acidic protein (GFAP) in plasma were examined at various time intervals. Bromine exposure resulted in abnormal cage behavior such as head hitting, biting and aggression, hypervigilance, and hyperactivity. An increase in plasma GFAP and brain 4-hydroxynonenal (4-HNE) content also was observed in the exposed animals. Acute and delayed sympathetic nervous system activation was also evaluated by assessing the expression of catecholamine biosynthesizing enzymes, tryptophan hydroxylase (TrpH1 and TrpH2), and tyrosine hydroxylase (TyrH), along with an assessment of catecholamines and their metabolites. TyrH was found to be increased in a time-dependent manner. TrpH1 and TrpH2 were significantly decreased upon Br_2_ exposure in the brainstem. The neurotransmitter content evaluation indicated an increase in 5-HT and dopamine at early timepoints after exposure; however, other metabolites were not significantly altered. Taken together, our results predict brain damage and autonomic dysfunction upon Br_2_ exposure.

## 1. Introduction

Bromine (Br_2_) is a volatile brownish-yellow fuming liquid. It has a strong pungent and suffocating odor that is irritating to the nasal mucosa. Br_2_ being three times denser than air with a value of 3.1028 g/cm^3^, tends to stay closer to the ground when released. Br_2_ is used extensively in manufacturing industries, agrichemicals, additives in leaded gasoline, dyes, and as a disinfectant in swimming pools. Consequently, it is stored and transported frequently, which increases the risk of accidental and malicious exposure. Exposure to Br_2_ may occur via inhalation, dermal, oral, or ocular routes. However, the major route of exposure to humans is via inhalation. Br_2_ on contact with aqueous media in the body forms bromide, highly oxidizing hydrobromic acid (HBr), hypobromous acid (HOBr), and bromic oxyacids (HBrO_3_). Br_2_ injury to humans is through direct irritant effect with the release of radical species that are damaging to the respiratory mucosa and also distant organs [1,2]. As low as 40–60 ppm is dangerous to human health, and cases of accidental spills with a high concentration of Br_2_ have been reported [3]. Its use in terror attacks has also been speculated [3]. Therefore, the understanding of its metabolism and effect on different organs of the body is of utmost importance as substantial gaps remain.

Br_2_ is widely distributed in extracellular tissue, and due to its highly reactive nature, it is converted into other moieties soon after contact [4]. We have demonstrated the occurrence of brominated fatty acids and fatty aldehydes, in the tissues of Br_2_-exposed animals, that form upon reaction of Br_2_ with pulmonary plasmalogens [2,5]. These circulate in the blood and are transported to other major organs in the body long after exposure [1]. Although not much is known regarding the metabolism of inhaled Br_2_, it has been known to form bromide, its anionic form, in living tissues [3,5]. Bromide is partitioned in the body similarly to chloride and acts by replacing chloride. It has been established that bromide forms a bactericidal oxidant under physiological concentration and plays a role in the myeloperoxidase system of neutrophils similar to chloride [6]. Bromide ion is a central nervous system (CNS) depressant, and its overdoses have adverse effects of their own [7]. Acute effects of Br_2_ on CNS have been found with the oral route of entry, while the inhalational route has been shown to cause chromodacryorrhea, upper respiratory symptoms, cough, headache, and lacrimation, along with CNS effects [3]. The injury of Br_2_ is not limited to the heart, lungs, skin, and eyes, but it has been seen that organic Br_2_-containing molecules are deleterious to CNS as well, regardless of the route of administration [3]. Headaches, dizziness, ataxia, slurred speech, and tremors vertigo are some of the symptoms seen in accidental Br_2_ exposure in human beings, and no specific treatments are available [3]. Nonetheless, limited toxicological effects of inhaled Br_2_ have been studied on the brain, but no biochemical studies have been performed hitherto. Hence, it is imperative to understand how Br_2_ would affect the biochemical parameters of the brain.

Using a rat model, our studies have shown that a single dose of acute Br_2_ exposure causes decreased heart rate, cardiac hypertrophy, increase in cardiac injury markers in blood, and increased ultrastructural damage [1,2]. We also demonstrated that the cardiac damage was caused by the presence of increased brominated fatty acids and aldehydes in the heart along with bromination of cardiac sarco(endo)plasmic reticulum calcium-ATPase (SERCA) and cardiac phosphalamban dephosphorylation that further decreased SERCA activity. We demonstrated that this SERCA inactivation was accompanied by increased cytosolic calcium (Ca^2+^) overload and increased Ca^2+^-sensitive LV protease (calpain) activity. These effects were followed by our group in the Br_2_-exposed rats for prolonged durations to observe the delayed effects. Interestingly, most of these effects persisted till 28 days of observation in the model and resulted in increased right ventricular (RV) and LV end-diastolic pressure and LV end-diastolic wall stress with increased LV fibrosis. The LV dysfunction in this model at this delayed duration was accompanied by SERCA inactivation that resulted potentially from increased oxidative stress, as shown by increased myocardial hydroxynonenal accumulation and increased NADPH oxidase-2 (Nox-2) [1]. SERCA2 inactivity could also be attributed to a striking loss of phosphalamban (PLN) phosphorylation that started 24 h after Br_2_ inhalation and persisted at 7, 14, and 28 days after exposure. Increased phosphatase 1 (PP1) was also demonstrated in this model, suggesting its possible contribution to PLN dephosphorylation. PP1 mediated PLN hypophosphorylation is critical to heart failure [8,9].

Thus, our studies established that the effects of inhaled Br_2_ might surpass the lungs and include the cardiovascular system. In the current study, we hypothesized that Br_2_ exposure causes the formation of the brominated moieties that possibly circulate to the brain via blood and causes brain damage via oxidative effects and modulation of the levels of neurotransmitters and their precursors. Behavioral changes observed also suggested a possibility of changes in their catecholamine metabolism in the brain. To test this hypothesis, we carried out experiments on rats exposed to Br_2_. Histological evaluation along with evaluation of markers of oxidative stress and injury to the brain and plasma were carried out. We specifically evaluated the brainstem as it contains the regions that control the respiratory center.

## 2. Results

### 2.1. Altered Cage Behavior Characteristics and Related Brain Injury Induced By Acute Br_2_ Exposure

**Figure 1:**
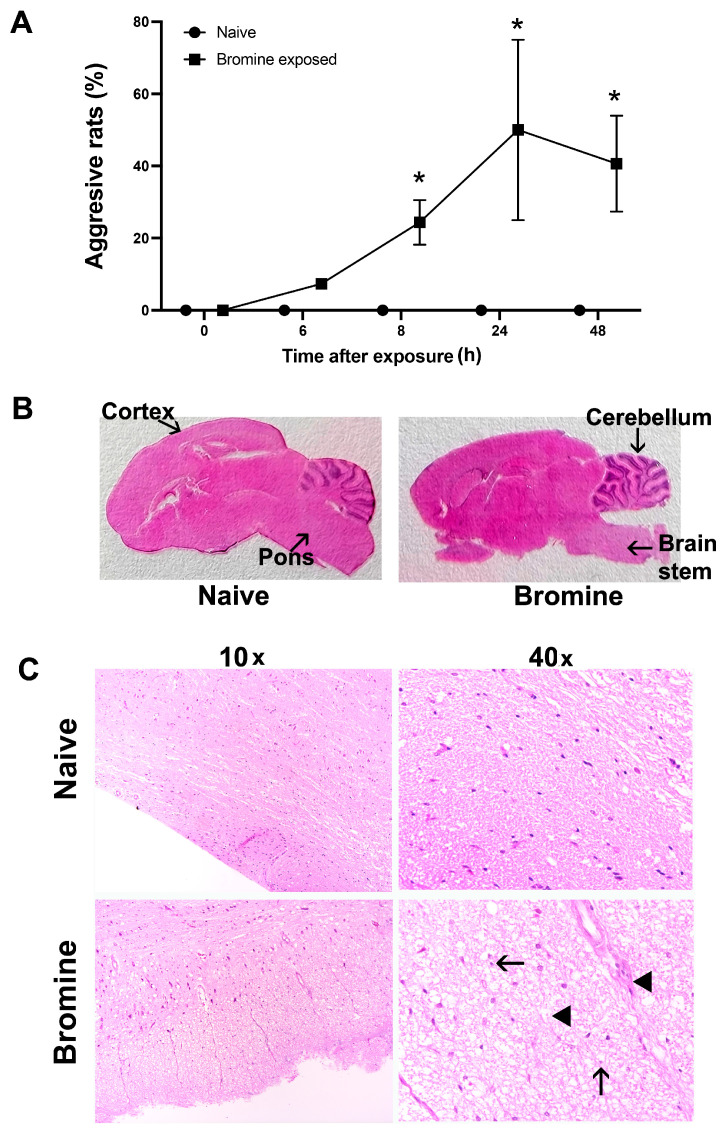
Bromine exposure impairs cage behavior and causes gross brain damage in rats. Sprague Dawley rats (male and female) were exposed to bromine (600 ppm for 45 min) and transferred to room air. Rats were monitored visually and for pulseoximetry and clinical symptoms every 2 h after exposure till 8 h and then at 24 h and 48 h. Cage behavior was qualitatively assessed by tabulating biting, head hitting, hyperactivity, and hypervigilance, as shown in (**A**). The data is expressed as Mean ± SEM (n = 8–12) with * *p* < 0.05. (**B**) Brain hematoxylin and eosin (H&E)-stained sections (1×) of the whole brain of naïve and Br_2_-exposed rats 24 h after exposure. Images were also collected of the brainstem area at 10× and 40× magnification, and representative images are shown in (**C**). The arrow demonstrates a shrunk apoptotic nucleus, and the arrowhead points to edematous spaces in the lower last panel of the bromine-exposed brainstem image (40×).

Behavioral aspects of halogen exposure are unknown. Br_2_-exposed rats exhibited peculiar cage behaviors, including hyperactivity and aggression, immediately following exposure. We observed that Br_2_-exposed rats exhibited frequent biting behavior towards cage mates, unstoppably chased cage mates, frequently engaged in head-hitting on cage walls, and were aggressive towards cage mates as well as towards experimenters handling rats. We scored the rats based on this aggression profile (bites, head hitting, hyperactivity, and hypervigilance). As mentioned in the methods section, rats with high aggression were given a score of 1, and rats that were docile were given a score of zero in both Br_2_-exposed and naïve animals. As depicted in Figure 1A, the percentage of rats exhibiting aggressive behavior increased with time in survivors after Br_2_ exposure and was maximum at 24 h. Next, we determined if an injury to the brain is the underlying cause of these behavioral changes.

Brain injury evaluated first with gross histological examination on brain sections of Br_2_-exposed rats at 24 h revealed global brain injury, which was more prominent in the brainstem, especially in the pons and cerebellar region as compared to naïve rats (Figure 1B). The H&E-stained brain sections of Br_2_-exposed rats also demonstrated histopathological changes such as ubiquitous apoptotic nuclei and edematous spaces, which were otherwise intact in similar regions of the brain in naive rats (Figure 1C).

### 2.2. Br_2_ Increases Brain Injury Biomarker GFAP and Elevates Oxidative Stress in the Brain Stem

**Figure 2:**
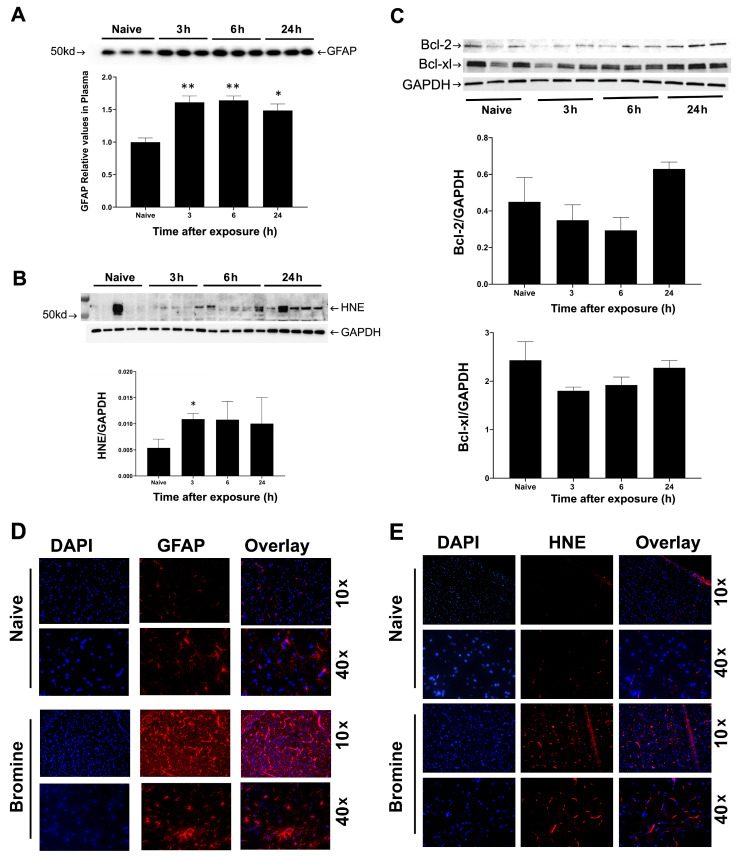
Bromine-induced brain injury and brainstem oxidative stress. (**A**) Rats were exposed to Br₂ at 600 ppm for 45 min and transferred to room air as described in the methods. Rats were euthanized at 3, 6, and 24 h, and blood and brain samples were collected. Immunoblots were performed and probed using anti-GFAP and 4-HNE antibodies. Circulating GFAP, a marker of acute brain injury, is demonstrated by a representative immunoblot with its respective densitometry below (**A**). The oxidative stress marker, 4-HNE in the brainstem homogenates, was also analyzed by western blot shown in panel B and densitometry as compared to the loading control shown below. GAPDH was used as the loading control (**B**). Apoptotic markers Bcl2 and Bcl-xl were also evaluated in the brainstem lysates by Western blot, as shown in (**C**). The data is represented as mean ± SEM (n = 3–5) with * *p* < 0.05, and ** *p*< 0.01. (**D**) Immunofluorescence staining was performed on the sagittal sections of the brain for GFAP and 4-HNE in bromine-exposed and naïve rats. Nuclear staining DAPI (blue) shown in the left panel with GFAP (Secondary antibody used was Alexa Fluor 594, as shown in the middle panel with composite images in the right panel. Similarly, in (**E**), DAPI-stained sections are shown in the left panel and 4-HNE stained in the middle panel and composite image in the right panel. Images were captured with a Leica Microscope (DM) at magnification 10×, 40× as labelled.

To evaluate the possible injury in the brain due to Br_2_, we evaluated GFAP (Glial fibrillary acidic protein), a brain injury marker [10]. We first determined plasma levels of GFAP at various timepoints to access brain injury by immunoblotting. Statistically significant increases in GFAP protein levels were observed starting at 3 h, which persisted till at least 24 h post Br_2_ exposure when compared to the naïve rats (Figure 2A). 4-HNE, which is a cytotoxic lipid peroxidation product, has been previously used as a marker for oxidative stress in brain injury of rats [11]. We observed an increase in 4-HNE in brain lysates at 3 (*p*-value < 0.05), 6, and 24 h in Br_2-_exposed rats as compared to naïve (Figure 2B). 4-hydroxynonenal is a lipid peroxidation product and an important mediator of oxidative brain injury and inducer of apoptosis in neural cells [12,13]. We also evaluated if cell death and apoptosis were occurring in the brain tissue after Br_2_ inhalation. Decreased levels of Bcl-2 and Bcl-xl proteins play an important role in brain injury [14]. The levels of Bcl-2 and Bcl-xl proteins were decreased at the earlier durations (3 h and 6 h) after exposure that we evaluated; however, the values were not significantly different from the controls (Figure 2C). We further confirmed the injury in the brain by performing immunofluorescence on the brainstem sections (IF). There was a marked increase in GFAP expression (Figure 2D) and 4-HNE (Figure 2E) in the Br_2_-exposed group when compared to the controls. We also evaluated if oxidative chemical modifications occur after bromine inhalation in the brainstem. Both protein tyrosine bromination and protein tyrosine nitration occurred 24 h after Br_2_ exposure (Figure S1).

### 2.3. Effect of Br_2_ Exposure on Aromatic Amino Acid Hydroxylases (AAAHs)

**Figure 3:**
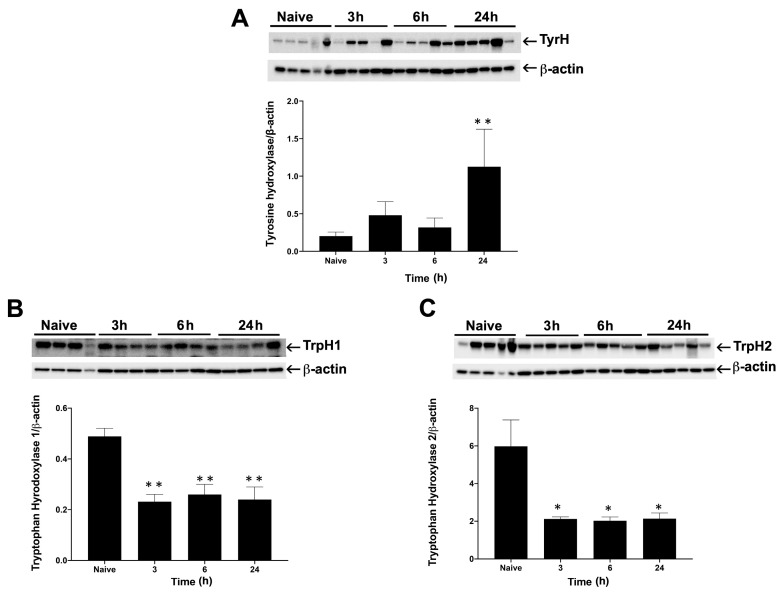
Tryptophan Hydroxylase (TrpH) and Tyrosine hydroxylase (TyrH) expression in brainstem after bromine exposure. Rats were exposed to bromine (Br₂) at 600 ppm for 45 min and brought to room air. Euthanasia was performed for necropsy at 3, 6, and 24 h, and brainstems were collected, and lysates were made for Western blots. Immunoblots and their respective densitometry analysis are demonstrated for tyrosine hydroxylase (TyrH) (**A**), tryptophan hydroxylase 1 (TrpH1) (**B**), and tryptophan hydroxylase 2 (TrpH2) (**C**). The loading control β-actin was used as shown under each blot. The data is represented as mean ± SEM (n = 4–5) with * *p* < 0.05, and ** *p* < 0.01.

**Figure 4:**
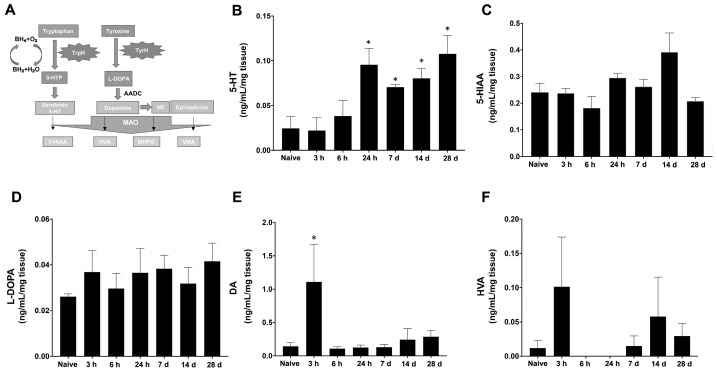
Bromine exposure in rats alters levels of precursors, metabolites, and neurotransmitters in the brainstem. (**A**) represents the schematic pathway of amino acids (tryptophan and tyrosine) conversion into neurotransmitters (serotonin and dopamine) and hormones (norepinephrine, epinephrine). LC-MS was performed on brainstem samples collected at various time intervals from naïve and Br_2_-exposed rats to assess the levels of 5-HT (Serotonin, **B**), 5-HIAA (5-hydroxyindoleacetic acid, **C**), L-DOPA, (**D**) DA (Dopamine; **E**), and HVA (Homovanillic acid, **F**). Data are represented as mean ± SEM (n = 4–6) with * *p* < 0.05.

Alterations in biopterin-dependent aromatic amino acid hydroxylases (AAAHs) are associated with many neurometabolic and neuropsychiatric disorders [15]. The aromatic amino acids are involved in the catalytic formation of neurotransmitters. To highlight the link between neuronal injury and neurotransmitters, we first evaluated the rate-limiting enzymes responsible for the formation of neurotransmitters. For that, we analyzed brainstem lysates of Br_2_-exposed and naïve rats. Tyrosine Hydroxylase (TyrH) is the major rate-limiting enzyme for hydroxylation of L-tyrosine to L-DOPA (Dihydroxyphenylalanine), which further converts into dopamine, epinephrine, and norepinephrine, the major adrenergic catecholamines. We found increased expression of TyrH at 3, 6, and 24 (*p* < 0.001) hours post-exposure of rats to Br_2_ as compared to the naïve group (Figure 3A)_._ Similarly, we investigated Tryptophan hydroxylase 1 (TrpH1; found in CNS and in other body parts) and TrpH2 (particularly neuronal), the two isoforms of the same enzyme [15]. TrpH1 and TrpH 2 are also rate-limiting enzymes in catalyzing the conversion of tryptophan to 5-hydroxytryptophan (5-HTP) and eventually to serotonin (5-HT) Figure 4A. TrpH 1 (*p* < 0.01) and TrpH 2 (*p* < 0.05) was significantly decreased 3, 6, and 24 h of exposure as compared to naïve (Figure 3B,C).

### 2.4. Br_2_ Exposure Alters Levels of Brain Stem Catecholamines

Catecholamines are responsible for many physiological functions, including cardiovascular, endocrinal, pulmonary, renal, and neurotransmission functions. Br_2_ is known to cause lung injury and alter lung function [16]. We assessed neurotransmitters following Br_2_ exposure by evaluating catecholamine in the brainstem that accommodates the respiratory center [15]. Figure 4A demonstrates the pathway for the precursors and the catecholamines, along with their respective enzymes and metabolites in the brain. Tryptophan, one of the essential amino acids involved in the formation of serotonin, and tryptophan hydroxylase, TrpH, is a rate-limiting enzyme essential for this conversion. 5-Hydroxyindoleacetic acid (5-HIAA) is the byproduct of serotonin. On the other hand, tyrosine is a non-essential amino acid and a precursor of major neurotransmitters L-DOPA, dopamine, epinephrine, and norepinephrine. TyrH is the major enzyme to mediate the conversion of tyrosine into L-DOPA, which converts into the major neurotransmitter dopamine. Homovanillic acid (HVA) is the metabolite of dopamine down the line. All these neurotransmitters are involved in neuronal communication and in mood stability [17]. The brainstems from unexposed and Br_2_-exposed rats were processed for performing HPLC (high-performance liquid chromatography) to evaluate these metabolites.

The results demonstrated an increase in the levels of serotonin (5-HT), one of the major hormones involved in mood fluctuation. Levels increased at 24 h and stayed elevated 28 days post single Br_2_ exposure as compared to the naïve group (Figure 4B). Interestingly, the 5-HT metabolite, 5-HIAA, remained unchanged at 24 h following Br_2_ exposure but slightly peaked at 14 days post-exposure, which was statistically not significant. (Figure 4C). Similarly, L-DOPA levels were not significantly altered in Br_2_-exposed animals. Although not statistically significant, we observed the greatest increase in dopamine levels at a very early phase after Br_2_ inhalation (3 h) and a slight increase at later timepoints (14 and 28 days), which never returned to control levels (Figure 4E). Furthermore, HVA, a major metabolite of dopamine, increased at 3 h and stayed higher than controls at 14 and 28 days post-Br_2_-exposure (Figure 4F).

## 3. Discussion

The current study was designed to evaluate neurotoxic effects of high dose (600 ppm) acute Br_2_ exposure in adult rats. Our data showed that rats exposed to Br_2_ had behavioral changes, which indicated neurological toxicity. Histopathological comparison of brain tissue of naïve and Br_2_-exposed rats revealed significant global brain tissue damage. Our results also demonstrated that these rats had significant increases in GFAP in plasma and 4-HNE (4-hydroxynonenal) in brain tissue. These are well-established biomarkers of brain injury [11,18]. Interestingly, Br_2_-exposed rats also had altered levels of the neurotransmitter serotonin. A trend towards an increase in HVA, which is a marker for metabolic stress, was also observed after bromine exposure [19]. Collectively, these results suggest the potential of Br_2_ as a neurotoxin that induces changes in behavior, neuro-histopathology as well as oxidative and metabolic stress in the brain.

Inhaled smoke and other pollutants, such as traffic air pollution, have been reported to cause neurological damage causing anxiety and cognitive decline [20]. Case studies of accidental Br_2_ exposure in the past have shown that exposed individuals endure neurological damage and exhibit clinical manifestations such as headache, ataxia, vertigo, visual disturbances, hallucination, delusion, and psychotic behavior [3]. However, acute brain damage due to Br_2_ exposure in experimental models has not been evaluated. The parallel between brain neurotransmitter/catecholamines increases and aggressive behavioral/clinical parameters is established [21]. Moreover, decreased antioxidant content and higher oxygen consumption makes the brain tissue very prone to oxidative stress [22,23,24,25]. Besides causing pulmonary toxicity, Br_2_ can cause possible distant organ injury [2,26]. Therefore, in this study, we focused on behavioral changes seen in these rats after Br_2_ exposure and evaluated any possible brain damage. Interestingly, Br_2_-exposed rats exhibited physical inactivity soon after exposure but became aggressive later. The neurobiological basis of Br_2_-induced aggression has never been studied in Br_2_-exposed rats. This pathological behavioral aggressiveness that was measured through a qualitative check in these rats exhibit sideways threats, chasing, attack bites, and the difficulty of the lab personnel to be able to take their clinical parameters due to their aggressive and defensive response. A disruption from normal behavior to an aggressive phenotype imbalance the adaptive behavior of these rats resulting in the disruption of their daily eating, drinking, and mating abilities [27]. Therefore, our understanding of this behavior and its neuropathological parameters after Br_2_ exposure is important.

Astrocytes are the non-neuronal glial cells present in the brain to maintain homeostasis. These cells express GFAP, a cytoskeletal protein, which is overly expressed in astrocytes upon brain insult. GFAP is clinically used along with other clinical parameters to determine brain injury and is a marker for neurological decline and damage and is also considered a determinant of mortality and morbidity [28]. GFAP can be quantified in blood and in brain tissue to determine brain injury [10]. Brain injury measured by GFAP release in plasma has been reported before for inhaled gasoline vapors in Sprague Dawley rats [29]. GFAP was also increased in the brain sections of H_2_S inhaled mice [30]. It is possible that Br_2_ is severely toxic as compared to H_2_S to the brain tissue and causes a release of GFAP in plasma.

Halogens, such as Br_2_, are highly reactive and form hypobromous acid (HOBr) and brominated fatty acids and aldehyde upon reaction with the moist pulmonary surface and the plasmalogens [2]. Hypobromous acid may further cause the oxidation of lipids and antioxidants [31]. Hypobromous acid may also react with protein components and cause the formation of bromotyrosines, as observed in our tissues [32]. Bromotyrosines are also considered markers of oxidative stress [32]. Oxidized lipids and proteins may disrupt cellular metabolism and signaling mechanisms by increasing the formation of proinflammatory cytokines, modifying gene expression, and increasing cellular apoptosis and necrosis. Increased cell membrane permeability, ATP depletion, and formation of protein aggregates are also observed as a consequence of neural oxidative stress [33]. Increased permeability of the blood–brain barrier allows the passage and accumulation of neurotoxic lipids, such as ceramides. Ceramides act by multiple mechanisms to increase oxidative stress and neuronal apoptosis [33,34].

The brain is also vulnerable to oxidative stress due to the large quantity of polyunsaturated lipids in neuronal membranes, which are particularly prone to oxidation by free radicals. 4-HNE, derived from lipid peroxidation of linolenic acids and arachidonic acids, is one of the powerful neurotoxic products. 4-HNE induces structural and conformational changes to a large variety of proteins involved in transportation, protein synthesis and stress response properties [22]. The significance of 4-HNE in cognitive dysfunction is underscored by the fact that patients with Alzheimer’s have elevated 4-HNE levels [35]. Additionally, previous studies have shown that 4-HNE increased in the hippocampus region of the brain in rats after inhaling ultrafine particles [36]. Similarly, the current study on rats shows an increase in 4-HNE in the brainstem after Br_2_ exposure suggesting oxidative stress/damage in the brain, and this was linked to the altered levels of catecholamines and neurotransmitters.

Catecholamines are hormones produced endogenously in the neurons and released in axons and dendrites as well as in peripheral tissues, such as the adrenal medulla. They have a key role in memory, learning, addiction, neuronal communication, autonomic functions, and behavioral regulation [37]. The disruption of dopamine, epinephrine (adrenaline), and nor-epinephrine (nor-adrenaline) have been implicated in brain trauma and insult [38]. Alterations of catecholamine levels are linked to oxidative stress and cellular dysfunction [38]. Catecholamines can act as antioxidants or prooxidants depending on their concentrations and amount of free radical source [39]. Catecholamines are generally antioxidants but, in the presence of transition metals, become prooxidants [39]. It was, however, recently shown that accumulation of catecholamine metabolites due to increased activity of an aromatic amino acids hydroxylase called tyrosine hydroxylase increased neuronal oxidative stress in mice [40].

Aromatic amino acid hydroxylases are a set of enzymes involved in the formation of these catecholamines. The disruption of these enzymes is directly related to alterations in catecholamines and hence neurometabolic and neuropsychiatric disorders [41]. TrpH1 and TrpH2 are enzymes involved in the formation of serotonin, a major monoamine signaling molecule responsible for mood and many psychiatric disorders of the brain [15]. TrpH1 makes circulating serotonin, while TrpH2 is mostly involved in the formation of neuronal serotonin mostly [15]. Although serotonin is known to be involved in the regulation of behavioral as well as psychological functions such as mood, sleep, gastric motility, and aggression, there is increasing interest in its role in immune cells, particularly T-cells [42]. Serotonin is found to have high uptake in the spleen, where immune cells are present in abundance [43]. Serotonin levels are decreased in anxiety and depression, and the treatment with selective serotonin reuptake inhibitors (SSRIs) is aimed at increasing its levels and is used routinely in clinical practice [43]. Serotonin has also been linked to neurotoxicity upon the use of inhalational anesthetics, where some anesthetics increased serotonin, leading to detrimental effects on the brain [44]. Our studies show a significant increase in the levels of serotonin along with a decrease in levels of its hydroxylases TrpH1 and TrpH2. Enhanced serotonin levels in our study could be related to a greater accumulation in the brain rather than synthesis. The increase in serotonin, possibly through negative feedback, may also decrease the expression of TryH1 and TryH2 enzymes, which are involved in the formation of serotonin. There was no significant increase in its metabolite, 5-HIAA. Tyrosine hydroxylase (TyrH) is one of the enzymes involved in the formation of dopamine and its precursor, L-DOPA. Dopamine is a neurotransmitter involved in many functions of the body, such as motor control, cognitive functions, reproduction behavior, and maternal instincts. Pathological conditions such as Parkinson’s, schizophrenia, Huntington’s, and attention deficit and hyperactivity disorder have altered levels of dopamine [45]. Increased oxidative stress, such as in smoking, has also been shown to increase levels of both dopamine and serotonin [46]. Likewise, our results show an increase in dopamine (acutely at 3 h) and its rate-limiting enzyme, TyrH. There is seemingly an increase in its precursor, L-DOPA, but the increase was not statistically significant. The increase in serotonin and dopamine could be linked to Br_2_-mediated neurotoxicity and inflammation [43]. HVA is a breakdown product of dopamine, epinephrine, and norepinephrine, mainly present in the peripheral system but is also in the central nervous system. HVA is believed to be a marker for metabolic stress in the brain and is also linked to exaggerated dopamine response to environmental stimuli in schizophrenia [19]. Our studies here demonstrated a trend towards an increase in HVA levels in the brain, indicating the onset of metabolic stress and hence an increase in dopaminergic molecules linking it to the disruption of behavioral (anxiety/aggression) and physiological measures. Our previous studies demonstrated a decrease in plasma catecholamines at acute timepoints measured at 15 min and 3 h post Br_2_ exposure [2].

**Figure 5:**
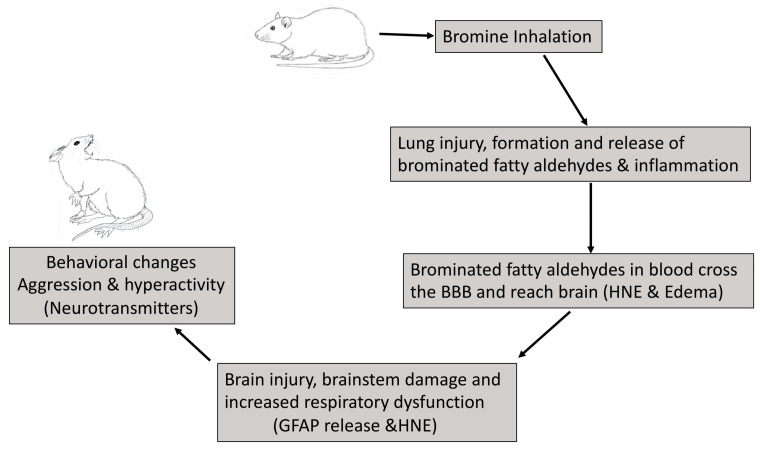
A schematic diagram of postulated events leading to behavioral and neuronal effects in surviving rats after bromine exposure. Inhalation of bromine causes inflammation of the lungs and the formation of reactive brominated amines and fatty acids and aldehydes that circulate to the brain and rest of the body. These reactive species cause brain injury and oxidative damage (4-HNE) and an alteration of the normal homeostasis of hormones and neurotransmitters, bringing behavioral changes (aggression).

Our observations in the present study, including cage behavioral changes, increase in oxidative and metabolic stress, along with an increase in catecholamines and stress hormones, epinephrine, and norepinephrine, suggest that Br_2_ upon inhalation causes brain damage secondary to pulmonary damage (Figure 5). We believe that it is through circulating brominated aldehydes generated following Br_2_ exposure which most likely contributes to the brain injury in rats. Biochemical and functional derangements of the brain and possible neurological disabilities due to single Br_2_ exposure have not been shown before. Although this study offers a novel understanding of increasing catecholamines and behavioral sequela of Br_2_ exposure, a detailed mechanistic study is warranted to better understand the neurological sequela of people with accidental or deliberate Br_2_ exposure to produce better therapeutic interventions. Careful mechanistic investigation into chemical, structural, and functional neuronal changes after bromine exposure is warranted.

## 4. Materials and Methods

### 4.1. Animals, Bromine Exposure and Cage Behavior Assessment

All animal studies were approved by the University of Alabama at Birmingham Institutional Animal Use and Care Committee (UAB IACUC #10282). Age-matched male and female Sprague Dawley rats were purchased from Envigo (Indianapolis, IN, USA). Whole-body bromine (600 ppm in air, 45 min) exposure was performed as described before [1]. These conditions model acute human exposures that occur during industrial or transport accidents [47]. Briefly, two unanesthetized rats (10–12 weeks of age and 250–300 g body weight) were exposed at a time in a whole-body Br_2_ exposure chamber housed in a specialized chemical fume hood in the exposure room of our facility for 45 min. Twelve rats (6 male and 6 female) per group (0 or 600 ppm) were used. Rats were then transferred to room air and observed for clinical symptoms, oxygenation, and qualitative behavioral assessment as described before [23]. Rats with aggression and the above-mentioned aspects were given a score of 1, and rats that were docile were given a score of zero in both Br_2_-exposed and naïve (0 ppm) animals. At the end of 48 h, arterial blood was collected from the descending aorta under isoflurane (2%–5% in room air) prior to exsanguination and subsequent necropsy. A separate group of rats (6 male and 6 female per group) were exposed to Br_2_ as described above and sacrificed at 3, 6, and 24 h for necroscopy. Arterial blood was collected from descending aorta under isoflurane anesthesia as described above. Separate animals were utilized for fixation of the organs and for freezing. Frozen tissues (brainstem) were used for subsequent bio/chemical analysis.

### 4.2. Histology and Immunofluorescence

For histology, tissues obtained at the study endpoint were processed for imaging following the previously described methods [2]. Image acquisition was performed on a Leica DM6000 epifluorescence microscope with Simple PCI software (Compix, Inc., Cranberry Township, PA, USA). For fixation, brain tissues were immersed in 10% neutral buffered formalin (NBF) and embedded in paraffin. Sections of 5 µm were cut and mounted on positively charged slides. For immunohistochemistry, deparaffinized and rehydrated brain tissue sections were then incubated with antigen unmasking solution (Vector Laboratories Burlingame, CA, USA #H3300) for 20 min for antigen retrieval. Nonspecific binding sites were blocked with 5% goat serum (in 1% bovine serum/phosphate-buffered saline, PBS), followed by overnight incubation (4 °C) with anti-HNE antibody 4-hydroxynonenal (4-HNE) (Millipore Burlington, MA, USA #393207; 1:200) or anti-GFAP (Abcam Cambridge, MA, USA, #ab7620; 1:200). The next day, tissue sections were incubated with secondary Alexa Fluor 594 conjugated anti-rabbit antibody (Life technologies Carlsbad, CA, USA, #A21207, dilution 1:1000) for 1 h at room temperature in a humid chamber. For the negative control, rabbit IgG control was used at the same specifications. Tissue sections were mounted using DAPI containing Vectashield mounting media (Vector labs, catalog #H1400-10). Images were acquired using a Leica Microscope (DM).

### 4.3. Western Blots

Brainstems were homogenized and assayed for protein levels. Immunoblots on plasma and brainstem lysates were performed according to previously described methods using antibodies against GFAP (Abcam Cambridge, MA, USA, #ab7620, 1:1000), Tyrosine hydroxylase (Millipore Burlington, MA, USA, AB152, 1:1000), Tryptophan hydroxylase 1 (ABclonal Technology Woburn, MA, USA, A1569, 1:1000), Tryptophan hydroxylase 2 (ABclonal Technology Woburn, MA, USA, A7147, 1:1000), GAPDH (1:5000, Cell Signaling Technology, Danvers, MA, USA), and 4-HNE (Millipore Burlington, MA, USA #393207, 1:1000) [2].

### 4.4. Neurochemical Analysis

Rat brainstem neurochemicals were analyzed in Dr. Rumbeiha’s laboratory using previously standardized protocols. To assess Br_2-_induced neurochemical changes, the brainstem was dissected out from the naïve and bromine-exposed rats and evaluated for changes in dopamine and its precursor 3, 4-dihydroxyphenylacteic acid (DOPA) and homovanillic acid (HVA). The brainstem was also analyzed for serotonin (5-HT) and its metabolite, 5-HIAA, and norepinephrine (NE). Samples were prepared and quantified as described previously [48]. Briefly, neurotransmitters were extracted from brainstems in 0.2 M perchloric acid solution containing 0.05% EDTA and 0.1% sodium metabisulfite and isoproterenol (internal standard). Dopamine serotonin and its metabolites were analyzed by HPLC, and data acquisition and analysis were performed using Chromeleon 7 and ESA Coularray 3.10 HPLC Software, ESA, Fort Collins, CO, USA [49].

### 4.5. Data Analyses

Data are expressed as mean ± standard error (SE) analyzed by one-way analysis of variance (ANOVA), and an unpaired Welch’s *t*-test was performed. A *p*-value < 0.05 was considered significant. All statistical analyses were conducted using Graphpad Prism version 9 software (La Jolla, CA, USA).

## Data Availability

Data is contained within the article.

## Supplementary Materials

The following are available at https://www.mdpi.com/article/10.3390/ijms22126316/s1.
