# Supplementary material for: Behavioral and Neuronal Effects of Inhaled Bromine Gas: Oxidative Brain Stem Damage
Source: Int J Mol Sci. 2021 Jun 12;22(12):6316. doi: 10.3390/ijms22126316 (PMC8231550; doi:10.3390/ijms22126316)
Supplement: Supplementary file 1 [file ijms-22-06316-s001.zip › ijms-1233364-supplementary.pdf]

Behavioral and Neuronal Effects of Inhaled Bromine Gas: Oxidative Brain Stem Damage

Shazia Shakil [1], Juan Xavier Masjoan Juncos [1], Nithya Mariappan [1], Iram Zafar [1], Apoorva Amudhan [1], Archita Amudhan [1], Duha Aishah [1], Simmone Siddiqui [1], Shajer Manzoor [1], Cristina M Santana [2], Wilson K Rumbeiha [3], Samina Salim [4], Aftab Ahmad [1] and Shama Ahmad [1,*]

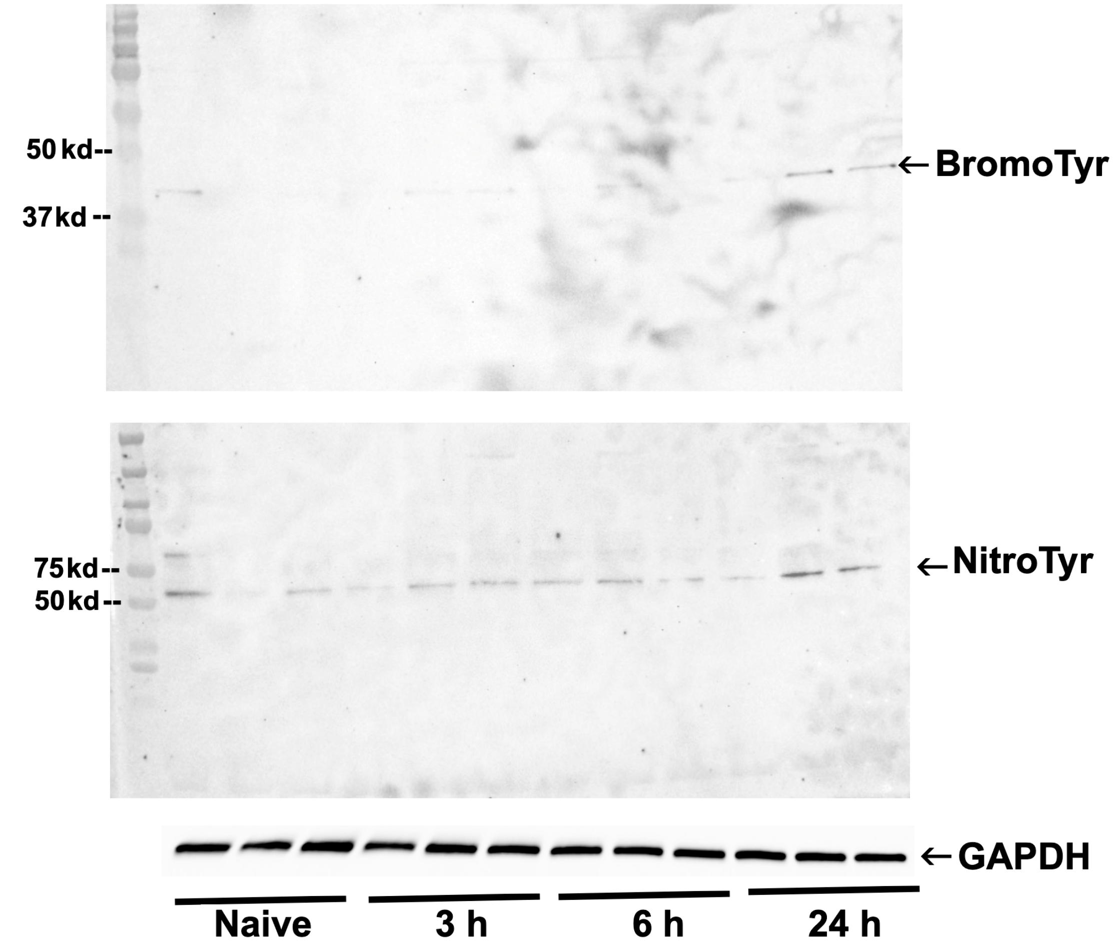

Figure S1. Bromine exposure causes chemical modification in the brainstem of rats. Rats were exposed to bromine as described in the Methods section and transferred to room air. Animals were sacrificed at different time intervals, brainstems were removed and lysates were prepared, and Western blots were performed. The top panel demonstrates the formation of bromotyrosines, and the bottom panel demonstrates nitrotyrosylation of proteins (around 50 kd mwt).
